# The Treatment of Constipation

**Published:** 1900-03

**Authors:** Joseph M. Mathews

**Affiliations:** Louisville, Ky., Professor of Surgery and Clinical Lecturer on Diseases of the Rectum: ex-President of the American Medical Association; President Kentucky State Board of Health, etc., etc.


President.

J. G. FIFE, Secretary.
Dallas, Texas.

### Abstracts and Selections.

### THE TREATMENT OF CONSTIPATION.

### A Clinical Lecture Delivered at the Hospital College of Medicine, Louisville, Kentucky.

BY JOSEPH M. MATHEWS, M. D., LL. D., LOUISVILLE, KY.,
Professor of Surgery and Clinical Lecturer on Diseases of the Rectum; ex-President of the American Medical Association; President Kentucky State Board of Health, etc., etc.

From the *New England Medical Monthly*, November, 1899.

GENTLEMEN: During the present session of the school, which is now nearing its close, you have witnessed operations for nearly every known disease of the rectum. I am sure that you are convinced now, if never before, of the absolute necessity of giving some special study to this class of affections. I trust, too, that by this clinical demonstration you will have been profited sufficiently to do many of these operations, thereby relieving a large class of sufferers, a class, too, which has been wonderfully neglected in the past by the profession. You know how common it is for all such affections to be designated as "piles," and the patient to be assured that an ointment will effect a cure. Your experience here will prove to you what an error it is to so classify these troubles. You have seen at these clinics men and women whose lives have been wrecked by the want of proper treatment. Need I mention such formidable diseases of the rectum and colon as tuberculosis, syphilis and cancer, or the so-called minor affections, as hemorrhoids, fistula, proctitis, ulceration, stricture, prolapse, polypoid growths, eczemas, pruritus, etc. Let me beseech you, therefore, not to look too lightly upon this class, but at least give them the benefit of a careful examination before you dismiss them. As the last clinic to be held this session I have summoned a number of patients who are not seriously ill, nor do they need any surgical operation. You see here some aged and some middle aged, while here to my right is a very young person. Each one of these is a subject of that very common, and, what is generally regarded, very simple ailment—*constipation*. Before I begin to explain the condition of these patients or this class of patients, permit me to say that constipation is a relative term. What is constipation to one is not constipation to another. Very often you will hear a person say, "If my bowels do not move every day I feel badly, headache, languor and tired." Another in apparent good health, will inform you that his or her bowels move only on every second, third or fourth day. The late Doctor D. W. Yandell once told me that a patient, in describing her trouble, said that so far as her bowels were concerned she was all right, as they moved with perfect regularity, *every two weeks*. I have made mention to you of a case treated by me and which is fully described in my work on Diseases of the Rectum, a young lady whose bowels moved only once every three months, four times a year.

I do not wish you to be impressed with the idea, either that constipation is a simple thing, for to the contrary, it is often a very serious affair. I once heard an old physician say that "if his bowels moved in the morning he was sure that he would not die that day." As he is now dead I have wondered "if his bowels moved that day."

Let us for a little time consider the physiology of defecation. The fæcal mass has the cæcum as its starting point, and when "a call of nature" takes place it means that a peristaltic wave occurs, which moves this mass rapidly through the colon, dropping it into the sigmoid flexure, thence into the rectum. If the "call" is heeded by the individual an "action" is the result. If, through false modesty, attention to business, or general laziness, attention is not paid to this effort of nature, then the watery constituent, which is the greater, is absorbed and carried into the circulation. In consequence we have an *auto-infection* which may prove of serious import. You can readily understand that by the absorption of the fæcal mass, a poison, that the whole general system would be deranged. The red corpuscles of the blood are diseased, altered in color and lessened in power. Hence a sallow complexion, dark rings under the eyes, cold extremities because of less supply of oxygen; lethargy due to vitiated blood and enfeebled corpuscles. The system is not nourished, hence the loss of flesh; the diseased blood circulates through the nervous system, and there is in consequence nervous depression—we might say *nervous exhaustion*—the pulse is slow and easily compressed; the organs of digestion and assimilation are lowered; there is loss of memory, no concentration of thought, and a great disposition to drowsiness. Notwithstanding that these patients are generally "sleepy," they are not relieved by sleep. All the functions are unsatisfactorily performed. If this condition is not relieved, disease and suffering must be the result. There is another phase of constipation that I would have you consider. We have stated that the contents of the fæcal mass is absorbed, the solid portion remains in the flexure and rectum. Daily and weekly this dried mass is added to, and in consequence we have the whole pelvic circulation deranged; external piles are produced, internal piles are made to bleed; atony of the coats of the bowel takes place, congestion, inflammation and ulceration may result. Truly, then, constipation is no "light" matter. What, then, shall we do for this condition? I once heard a doctor say that he would give a thousand dollars for a "specific" for constipation. I really believe the investment would have been a good one, when we consider how many people are thus affected.

Before attempting to map out any line of treatment I wish to impress upon you that you should diagnosticate between what is known as *obstipation* and *constipation*. The former may arise from a mechanical cause, as an irritable and contracted spinchter, a stricture or growth in the rectum, and some believe that the valves of the rectum play a part here. Of course, if either one of these conditions is detected you should turn your attention to their removal, for the obstipation is only secondary to them. I have relieved many cases of so-called constipation by dilating the sphincter muscle. But what should be done in a medical way to eradicate this condition? Let me say that you will find as most excellent adjuvants in the treatments of many of these patients: electricity, massage of the abdomen, cold baths and exercise. Every physician seems to have some favorite prescription, in the form of pill or solution, but they are constantly informed that "they have lost their power." Of course you have heard that the "regular habit" should be indulged in; that enemas are good under certain conditions, and a pill is necessary. But do such effect a cure? Very rarely. Each case must be studied as an individual one. Fat people as well as lean are affected in this way—the young as well as the old. Women are more given to the habit than men, and I believe the reason to be that they are possessed of a womb. You will often find that a displaced uterus, or an enlarged one with adhesions, is responsible for the constipated condition. It is common with young school girls, who in the rush to get early to school neglect the very important duty of having their bowels move in the early morning. Among the serviceable drugs in the treatment of this affection you will find the following: cascara sagrada, sulphur, belladonna, nux vomicæ, sulph. iron, buckthorn, ipecac, magnesia, the mineral waters, and many others either alone or in combination.

But let me impress upon you the necessity of making a more thorough study of such a case. If the patient who consults you is really desirous of getting well he should at least give you a fair chance to cure him. Supposing then that you have such consent, I would advise you to proceed in the following way: First try and ascertain what is the cause of the constipation. In this connection I wish to state that after an examination and observation of these cases extending over twenty years, I am forced to believe that the majority of them have as a basis a constitutional derangement. In trying to solve the problem, it was observed that many of these patients were of a rheumatic or gouty diathesis. Acting upon this hypothesis, I have treated them by combating this special trouble and have found that in many cases the constipation would take care of itself. There are many preparations that you can use for this purpose, but the best is some form of lithia. Waters containing this salt will be found of service if taken in large quantities and for a long period of time. However, in my own practice I prefer to use the drug in a more concentrated form. I have, therefore, been using for some time a preparation of lithia known as thialion, with a marked degree of success. I direct that it be taken in teaspoonful doses, given in a full glass of hot water, taken before each meal. My theory is that in the rheumatic or gouty subject the intestines are brought under the same conditions that the disease or diseases are made manifest in other portions of the body. The muscular coat of the intestines is particularly affected by this gouty condition, and in consequence loses its contractile power. Anyway, I have cured patients of the confirmed constipation habit by this drug alone. To proceed, I would say to the patient that he must submit to my directions. You will find that in lieu of the rectal enema, that if a *high* enema is given through a Wales bougie say of a half to a gallon of water two or three times a week it will be much more satisfactory. The object is to replace the amount of water which has been lost by absorption of the fæces. A fruit diet, together with the drinking of large quantities of water should be enjoined. Massage of the abdomen by the patient himself, who should be taught the route of the colons, should be advised. The sweets should be forbidden and only plain, nutritious diet observed. I consider the administration of drastic purgatives harmful rather than beneficial. If you will watch this class of patients as carefully as you would any other chronic one, you will be awarded by success. I beseech you not to get into the habit of prescribing for them in a routine way, for if you do they will soon desert you, and go elsewhere, besides you will do them no good.

